# Fermentation weight loss, fermentation quality, and bacterial community of ensiling of sweet sorghum with lactic acid bacteria at different silo densities

**DOI:** 10.3389/fmicb.2022.1013913

**Published:** 2022-11-14

**Authors:** Haiwen Xu, Nier Wu, Na Na, Lin Sun, Yi Zhao, Haijun Ding, Yongyu Fang, Tianwei Wang, Yanlin Xue, Jin Zhong

**Affiliations:** ^1^College of Foreign Languages, Inner Mongolia University of Finance and Economics, Hohhot, China; ^2^Inner Mongolia Key Laboratory of Microbial Ecology of Silage, Inner Mongolia Engineering Research Center of Development and Utilization of Microbial Resources in Silage, Inner Mongolia Academy of Agriculture and Animal Husbandry Science, Hohhot, China; ^3^Institute of Microbiology, Chinses Academy of Science, Beijing, China

**Keywords:** bacterial community, bacterial diversity, fermentation weight loss, microbial counts, nutritional compositions, organic acid, sweet sorghum silage

## Abstract

Sweet sorghum is an important forage in arid and semi-arid climatic regions. This study aimed to reveal the fermentation weight loss (FWL), fermentation quality, and bacterial community of ensiling of sweet sorghum with lactic acid bacteria LAB; (*Lactiplantibacillus plantarum* and *Lentilactobacillus buchneri*) at different silo densities. For this study, sweet sorghum was harvested at the first spikelet of inflorescence stage and ensiled without or with LAB (CK or L) in polyethylene laboratory-scale silos (diameter, 20 cm; height, 30 cm) at densities of 650 (CK_650 and L_650), 700 (CK_700 and L_700), and 750 kg/m^3^ (CK_750 and L_750), respectively. The FWL, fermentation quality, microbial counts, and bacterial community of the silage were assessed after 100 days of ensiling. L_750 had a lower FWL than CK_650, _700, and _750 after 100 days of ensiling (*P* < 0.005), and the FWL was affected by silo density and inoculating LAB (*P* < 0.005). All silages had low pH (<4.0) and ammonia nitrogen content (<50 g/kg total nitrogen) and did not contain propionic and butyric acids; moreover, inoculating LAB increased lactic and acetic acids (*P* < 0.005). Bacterial communities in inoculated and uninoculated silages were clustered together, respectively, and clearly separated from each other. The total abundance of *Lactiplantibacillus* and *Lentilactobacillus* in fresh forage was <1%. *Lactiplantibacillus* had the highest abundance in all silages (from 71.39 to 93.27%), followed by *Lentilactobacillus* (from 3.59 to 27.63%). Inoculating LAB increased the abundance of *Lentilactobacillus* in each silo density (*P* < 0.005) and decreased *Lactiplantibacillus* in the silage in densities of 700 and 750 kg/m^3^ (*P* < 0.005); moreover, increasing silo density decreased *Lactiplantibacillus* abundance and increased *Lentilactobacillus* abundance in inoculated silages (*P* < 0.005). Overall, sweet sorghum silage showed satisfactory fermentation quality, with a density of no <650 kg/m^3^, and inoculating LAB improved fermentation quality and reduced FWL. *Lactiplantibacillus* and *Lentilactobacillus* presented as minor taxa in fresh sweet sorghum and dominated the bacterial community of all silages. Inoculating LAB was the main factor affecting the bacterial community of sweet sorghum silage. Moreover, inoculating LAB and increasing silo density can contribute to the decreasing *Lactiplantibacillus* abundance and increasing *Lentilactobacillus* abundance.

## Introduction

Sweet sorghum is an important crop in arid and semi-arid climatic regions. It can tolerate adverse environments such as limited rainfall, high temperature, and low soil fertility (Amer et al., 2012). This tolerance can be attributed to their primary roots, which absorb water and nutrients from the soil, and the secondary roots, which penetrate very deep into the soil (Tudisco et al., 2021). In addition to its use as a bioenergy crop, sweet sorghum has the potential to be used as forage for livestock (Orrico et al., 2020). The presence of a high concentration of water-soluble carbohydrates (WSCs) in sweet sorghum improves its ensilability when inoculated with more lactic acid bacteria (LA) during the fermentation process (Adesogan et al., 2004; Orrico et al., 2020). Therefore, ensiling is a satisfactory method for preserving sweet sorghum to provide high-quality forage to livestock production all year round (Orrico et al., 2020). Previous studies showed that sweet sorghum silage can replace corn silage in the dairy cow TMR without any negative effect on milk production and quality (Colombini et al., 2012; Tudisco et al., 2021), and the production performance of sheep can be sustained by feeding sorghum silage in replacement of corn silage (Sabertanha et al., 2021).

In recent years, the interest in sweet sorghum silage had increased, with greater prominence in areas where water resources are in short supply (Fernandes et al., 2020). Most previous studies showed the satisfactory fermentation quality detected in sweet sorghum silage without any treatment (Li et al., 2017; Sifeeldein et al., 2018; Alhaag et al., 2019; Orrico et al., 2020; Diepersloot et al., 2021). Inoculating lactic acid bacteria (LAB) for ensiling of sweet sorghum improves fermentation quality by reducing pH and ammonia nitrogen (AN) and increasing LA of the silage (Li et al., 2017; Sifeeldein et al., 2018; Alhaag et al., 2019). However, some studies reported that microbial inoculants have no positive effect on the fermentation quality of sweet sorghum silage (Orrico et al., 2020; Diepersloot et al., 2021). Ren et al. (2021) showed that ensiling of sweet sorghum with rumen fluid improves the fermentation quality of terminal silage, and *Lactobacillus* is the predominant bacterial community used for ensiling. Moreover, *Lentilactobacillus buchneri* and *Lentilactobacillus hilgardii* are predominant bacterial genera used in co-ensiling of *Sesbania cannabina* and sweet sorghum with LAB inoculants (Xia et al., 2022). However, there is limited information about the effect of silo density on fermentation weight loss (FWL), fermentation quality, and bacterial community of sweet sorghum silage in the literature.

We hypothesized that ensiling of sweet sorghum with LAB inoculants at different silo densities can alter the FWL, fermentation quality, and bacterial community of the silage. Thus, the objectives of this study were to determine the dynamics of FWL during the fermentation process, as well as the fermentation quality and the bacterial community of the terminal silage.

## Materials and methods

### Preparing silages and sampling

For this study, sweet sorghum [*Sorghum dochna* (Forssk.) Snowden] was grown on an experimental farm of Inner Mongolia Academy of Agriculture and Animal Husbandry Science, Hohhot, China. It was harvested at the first spikelet of inflorescence stage from four different fields as replicates on 26 September 2021. The fresh forages from each field were separately chopped into 1- to 2-cm pieces, mixed thoroughly, and then divided into two batches: One batch (CK) was sprayed with 10.0 ml/kg fresh weight (FW) of distilled water, and the other batch (L) was sprayed with 10.0 ml/kg FW of distilled water and 5 g/t FW (recommended amount) of commercial LAB additives (*Lactiplantibacillus plantarum* 550 and 360 (≥1.3 × 10^10^ CFU/g) and *Len. buchneri* 225 (≥7.0 × 10^9^ CFU/g); Zhuanglemei. Sichuan Gaofuji Biotechnology Co. Ltd, Chengdu, China). After thorough mixing, the CK batch from each field was divided into three sub-batches for the three silo densities, and the three sub-batches were packed into three polyethylene laboratory-scale silos (diameter, 20 cm; height, 30 cm) at densities of 650 (CK_650), 700 (CK_700), and 750 kg/m^3^ FW (CK_750), respectively. The L batch from each field was divided into three sub-batches for the three silo densities, and the three sub-batches were also packed into three polyethylene laboratory-scale silos (diameter, 20 cm; height, 30 cm) at densities of 650 (L_650), 700 (L_700), and 750 kg/m^3^ FW (L_750), respectively. The 24 silos (6 treatments ^*^ 4 replicates) were stored at ambient temperature (22–25°C) and sampled at 100 days of ensiling to determine the fermentation quality, microbial counts, bacterial community, and nutritional compositions.

### Fermentation weight loss

The FWL was recorded for 24 silos following the method mentioned in Samarasinghe et al. (2019). The weights of the silage silos were measured at 0, 1, 3, 6, 15, 30, 50, and 100 days of ensiling, and the weights of silos before ensiling were also measured.


$$\begin{array}{l} \text{ FWL at }x\text{ d }\left(\frac{\text{g}}{\text{kg}}\text{FW}\right)\\ =\;\frac{\text{Weight of silage silo at 0 d }-\text{ Weight of silage silo at }x\text{ d}}{\text{Weight of silage silo at 0 d }-\text{ Weight of silo before ensiling}}\\ \;\times 1000 \end{array}$$

*x d* = 1, 3, 6, 15, 30, 50, and 100 days of ensiling.

### Fermentation quality

The fresh forages or silages were dried at 65°C for 48 h using a forced-air oven (BPG-9240A, Shanghai Yiheng Scientific Instrument Co., Ltd., Shanghai, China) to measure the dry matter (DM) content, and then were ground through a 1-mm screen using a mill (FS-6D; Fichi Machinery Equipment Co., Ltd., Shandong, China) for measuring buffering capacity (BC) and nutritional components.

The extract was prepared from fresh forage or silage (25 g) and homogenized with sterile water (225 ml) for 100 s by using a flap-type sterile homogenizer (JX-05, Shanghai Jingxin Industrial Development Co., Ltd., Shanghai, China) and filtered through four layers of cheesecloth (Xu et al., 2021). The pH of fresh forage or silage was assessed using a pH meter (PB-10, Sartorius, Gottingen, Germany) to measure the extract. After filtrating through a filter membrane (0.22 μm), the concentrations of LA, acetic acid (AA), propionic acid, and butyric acid in silage were assessed by high-performance liquid chromatography (DAD, 210 nm, SPD-20A, Shimadzu Co., Ltd., Kyoto, Japan). The conditions were as follows: detector, SPD-20A diode array detector, 210 nm; column, Shodex RSpak KC-811, 50°C (Showa Denko K.K., Kawasaki, Japan); and mobile phase, 3 mM HClO_4_, 1.0 ml/min (Wang et al., 2021). The AN concentration in silage was assessed using a Kjeltec autoanalyzer (8400; Foss Co., Ltd., Hillerød, Denmark) according to the Kjeldahl method (AOAC International, 2005). The BC in fresh forage or silage was assessed using acid–base titration to measure the powder sample (Playne and McDonald, 1966).

### Microbial counts and bacterial community

The microbial counts in fresh forage or silage were assessed as described in Cai (1999). Coliforms, aerobic bacteria, and yeasts were cultured on violet red bile agar, nutrient agar, and potato dextrose agar, respectively, in an incubator (LRH-70, Shanghai Yiheng Science Instruments Co., Ltd, Shanghai, China) at 30°C for 72 h. Moreover, LAB were cultured on Man, Rogosa, and Sharpe agar under anaerobic conditions in the same incubator at 30°C for 72 h.

The bacterial DNA in the fresh forage or silage was extracted by using an E.Z.N.A. ^®^Stool DNA Kit (D4015, Omega Bio-tek, Inc., GA, USA) in accordance with the manufacturer's instructions. The V3–V4 region of the bacterial rRNA gene was amplified using a polymerase chain reaction (PCR) with primers 341F (5′-CCTACGGGNGGCWGCAG-3′) and 805R (5′-GACTACHVGGGTATCTAATCC-3′). The amplifying condition was as follows: 98°C for 30 s, followed by 32 cycles of denaturation at 98°C for 10 s, annealing at 54°C for 30 s, and extension at 72°C for 45 s, followed by a final extension at 72°C for 10 min (Logue et al., 2016). The PCR products were purified by using AMPure XT beads (Beckman Coulter Genomics, Danvers, MA, USA) and then quantified by using a Qubit Fluorometer (Invitrogen, USA). The purified and quantified PCR products were sequenced by an Illumina NovaSeq PE250 platform in accordance with the manufacturer's recommendations, provided by LC-Bio (Hangzhou Lianchuan Biotechnology Co., Ltd, Hangzhou, China). The paired-end reads were merged by FLASH. Principal coordinates analysis (PCoA) and bacterial community differences between CK_650, _700, and _750; L_650, _700, and _750; CK_650 and L_650; CK_700 and L_700; and CK_750 and L_750 were analyzed using R 3.6.1. Sequencing data were submitted to the NCBI Sequence Read Archive database (accession number: PRJNA860017).

### Nutrition compositions

The total nitrogen (TN) in fresh forage or silage was assessed by using a Kjeltec autoanalyzer (8400; Foss Co., Ltd., Hillerød, Denmark) with copper as the catalyst according to the Kjeldahl method, and the TN multiplied by 6.25 was the crude protein (CP) concentration in silage. The neutral detergent fiber (NDF) and acid detergent fiber (ADF) concentrations were assessed using an Ankom fiber analyzer (2000, Ankom, Macedon, NY, USA) without heat-stable amylase (Van Soest et al., 1991). The ash concentration in fresh forage or silage was assessed according to AOAC (2005).

### Statistical analyses

The data regarding the FWL were as a 3 × 2 × 7 factorial treatment structure. The model included three silo densities (650, 700, and 750 kg/m^3^ FW), two inoculating LAB levels (0 and 2 g/t FW), seven ensiling times (1, 3, 6, 15, 30, 50, and 100 days of ensiling), and their interaction (silo density ^*^ inoculating LAB, silo density ^*^ ensiling time, inoculating LAB ^*^ ensiling time, and silo density ^*^ inoculating LAB ^*^ ensiling time). The differences among seven ensiling times for each treatment and among six treatments for each ensiling time were analyzed using the GLM procedure of SAS (SAS System for Windows, version 9.1.3; SAS Institute Inc., Cary, NC, USA). The data regarding the fermentation quality, microbial counts, sequencing data, alpha diversity, and nutrition compositions were analyzed as a 3 × 2 factorial treatment structure. The model included three silo densities (650, 700, and 750 kg/m^3^ FW), two inoculating LAB levels (0 and 2 g/t FW), and their interaction (silo density ^*^ inoculating LAB). The differences among six treatments were also analyzed using the GLM procedure of SAS. The correlation heatmap between main bacterial genera and fermentation quality was built by R 3.6.1.

## Results

### Characteristics of materials

The characteristics of sweet sorghum before ensiling are presented in Table 1.

**Table 1:** Characteristics of sweet sorghum before ensiling (*n* = 4).

**pH**	**Microbial counts (log colony-forming units/g fresh weight)**				**Dry matter (DM, g/kg)**	**Nutrition compositions (g/kg DM)**					**BC** **(mE/kg DM)**
	**Lactic acid bacterial**	**Coliforms**	**Bacterial**	**Yeasts**		**CP**	**WSC**	**NDF**	**ADF**	**Ash**	
5.82	4.08	5.62	6.65	6.20	272	61.4	327	380	225	54.8	227

CP, crude protein; WSC, water-soluble carbohydrates; NDF, neutral detergent fiber; ADF, acid detergent fiber; BC, buffering capacity.

### Fermentation weight loss of silages during fermentation

The FWL in all treatments increased during the fermentation process (*P* < 0.05) (Table 2). The L_750 had the lowest FWL among all treatments from 15 to 100 days of storage (except CK_750 at 15 days and L_650 and _700 at 100 days) (*P* < 0.05). Moreover, CK_750 had lower FWL than CK_650 and _700 at 30 days (*P* < 0.05), and CK_750 and L_700 had lower FWL than CK_700 and CK_650 at 50 days, with FWL of L_650 lower than that of CK_650 (*P* < 0.05). Silo density, inoculating LAB, and ensiling time had a significant effect on the FWL of silages, which were interacted by silo density and inoculating LAB, and inoculating LAB and ensiling time (*P* < 0.05).

**Table 2:** Fermentation weight loss (% based on fresh weight) of sweet sorghum silages during the fermentation process (*n* = 4).

**Items**	**Ensiling days**							**SEM**	***P*** **-value**
	**1 day**	**3 days**	**6 days**	**15 days**	**30 days**	**50 days**	**100 days**		
CK_650	0.008f	0.052f	0.135e	0.383Ad	0.588Ac	0.964Ab	1.46Aa	0.016	<0.001
L_650	0.039f	0.101ef	0.171e	0.367Ad	0.549ABc	0.903BCb	1.38ABa	0.030	<0.001
CK_700	0.010f	0.055f	0.139e	0.378Ad	0.583Ac	0.942ABb	1.43Aa	0.017	<0.001
L_700	0.035f	0.104ef	0.178e	0.377Ad	0.551ABc	0.884Cb	1.37ABa	0.030	<0.001
CK_750	0.008g	0.049f	0.118e	0.325ABd	0.511Bc	0.858Cb	1.43Aa	0.010	<0.001
L_750	0.010f	0.048f	0.109e	0.291Bd	0.455Cc	0.794Db	1.27Ba	0.014	<0.001
SEM	0.016	0.026	0.024	0.016	0.012	0.015	0.032		
*P*-Value	0.532	0.438	0.309	0.008	<0.001	<0.001	0.013		
Interaction	D	L	T	D*L	D*T	L*T	D*T*L		
*P*-value	<0.0001	0.0014	<0.0001	0.0368	0.1303	<0.0001	0.9963		

CK, ensiling of sweet sorghum with 2.00 ml/kg fresh weight (FW) of distilled water at 650 (CK_650), 700 (CK_700), and 750 kg/m^3^ (CK_750) of density, respectively; L, ensiling of sweet sorghum with 2.00 g/t FW of lactic acid bacteria (LAB) inoculant and 2.00 ml/kg FW of distilled water at 650 (L_650), 700 (L_700), and 750 kg/m^3^ (L_750) of density, respectively. Values with different lowercase letters (a, b, c, d, e, f, and g) indicate significant differences among ensiling days for the same treatment (P < 0.05). Values with different uppercase letters (A, B, C, and D) indicate significant differences among treatments on the same day (P < 0.05). D, silo density; L, inoculating LAB; T, ensiling time.

### Fermentation quality of silages

The pH in L_650, CK_700, and L_750 was lower than that in CK_650 but higher than that in L_700 and CK_750 (*P* < 0.05) (Table 3). L_650, _700, and _750 had higher LA and AA concentrations and lower LA/AA than CK_650, _700, and _750 (*P* < 0.05). The AN in L_750 was higher than that in L_650 and CK_700 and _750, with AN in CK_750 lower than that in CK_650 (*P* < 0.05). CK_650, _700, and _750 had higher BC than L_650, _700, and _750, respectively, with BC in CK _700 higher than that in CK_650 and _750, and BC in L_700 higher than that in L_650 and _750 (*P* < 0.05). The pH was mainly affected by silo density and interacted by silo density and inoculating LAB (*P* < 0.05). The inoculating LAB had a significant effect on the LA and AA concentrations and LA/AA (*P* < 0.05). The AN was interacted by silo density and inoculating LAB (*P* < 0.05). The BC was mainly affected by silo density and inoculating LAB (*P* < 0.05).

**Table 3:** pH, organic acid concentrations [g/kg dry matter (DM)], ammonia nitrogen/total nitrogen (AN, g/kg), and buffering capacity (BC, mE/kg DM) in sweet sorghum silages (*n* = 4).

**Items**	**pH**	**LA**	**AA**	**LA/AA**	**AN**	**BC**
CK_650	3.29a	50.4b	7.75b	7.24a	31.7ab	531bc
L_650	3.27b	102a	36.4a	2.86b	27.7bc	517d
CK_700	3.26b	52.9b	8.55b	6.77a	27.1bc	546a
L_700	3.24c	96.1a	39.3a	2.47b	28.5abc	534b
CK_750	3.25c	56.3b	7.46b	7.98a	25.5c	526c
L_750	3.27b	114a	42.7a	2.68b	33.3a	516d
SEM	0.005	8.12	2.74	0.754	1.34	1.78
*P*-Value	<0.001	<0.001	<0.001	<0.001	0.006	<0.001
D	<0.001	0.367	0.585	0.634	0.212	<0.001
L	0.203	<0.001	<0.001	<0.001	0.077	<0.001
D*L	<0.001	0.664	0.523	0.755	<0.001	0.556

CK, ensiling of sweet sorghum with 2.00 ml/kg fresh weight (FW) of distilled water at 650 (CK_650), 700 (CK_700), and 750 kg/m^3^ (CK_750) of density, respectively; L, ensiling of sweet sorghum with 2.00 g/t FW of lactic acid bacteria (LAB) inoculant and 2.00 ml/kg FW of distilled water at 650 (L_650), 700 (L_700), and 750 kg/m^3^ (L_750) of density, respectively. Values with different lowercase letters (a, b, c, and d) indicate significant differences among treatments (P < 0.05); LA, lactic acid; AA, acetic acid. D, silo density; L, inoculating LAB.

### Microbial counts of silages

L_650 had a higher LAB count than CK_650 and _750 (*P* < 0.05) and a higher yeast count than CK_650, 700, and 750 (*P* < 0.05) (Table 4). CK_750 had a lower yeast count than L_650, 700, and 750 (*P* < 0.05). Coliforms were not detected in all silages. Inoculating LAB mainly affected the LAB and yeast counts (*P* < 0.05).

**Table 4:** Microbial counts (log colony-forming units/g fresh weight) in sweet sorghum silages (*n* = 4).

**Items**	**LAB**	**Bacterial**	**Yeasts**
CK_650	7.20bc	3.12	7.39bc
L_650	7.98a	3.55	7.84a
CK_700	7.33abc	3.04	7.34bc
L_700	7.46abc	3.62	7.64ab
CK_750	6.83c	3.59	7.17c
L_750	7.62ab	3.46	7.65ab
*P*-value	0.003	0.090	0.003
SEM	0.163	0.167	0.105
D	0.116	0.453	0.159
L	<0.001	0.056	<0.001
D*L	0.101	0.135	0.666

CK, ensiling of sweet sorghum with 2.00 ml/kg fresh weight (FW) of distilled water at 650 (CK_650), 700 (CK_700), and 750 kg/m^3^ (CK_750) of density, respectively; L, ensiling of sweet sorghum with 2.00 g/t FW of lactic acid bacteria (LAB) inoculant and 2.00 ml/kg FW of distilled water at 650 (L_650), 700 (L_700), and 750 kg/m^3^ (L_750) of density, respectively. Values with different lowercase letters (a, b, and c) indicate significant differences among treatments (P < 0.05). LA, lactic acid; AA, acetic acid; D, silo density; L, inoculating LAB.

### Bacterial community of silages

The fresh materials had higher observed OTUs and indexes of Shannon, Simpson, Chao1, and Pielou e than all silages (*P* < 0.05) (Table 5). L_650, _700, and _750 had a higher Simpson index than other treatments (*P* < 0.05), and L_700 and _750 had a higher Pielou e index than CK_750 (*P* < 0.05). Inoculating LAB mainly affected Shannon, Simpson, and Pielou e indexes (*P* < 0.05).

**Table 5:** Sequencing data and alpha diversity of bacteria in sweet sorghum silages (*n* = 4).

**Items**	**Raw tags**	**Valid tags**	**Observed OTUs**	**Shannon**	**Simpson**	**Chao1**	**Goods coverage**	**Pielou e**
Fresh	81419	76978	117a	3.97a	0.866a	118a	1.00	0.578a
CK_650	72473	70565	39.5b	1.01b	0.301c	39.8b	1.00	0.190bc
L_650	86390	81365	41.0b	1.16b	0.396b	41.9b	1.00	0.216bc
CK_700	77372	75296	44.5b	1.04b	0.291c	45.5b	1.00	0.191bc
L_700	85413	78715	47.0b	1.33b	0.467b	48.5b	1.00	0.242b
CK_750	81136	79341	45.3b	0.947b	0.254c	45.8b	1.00	0.173c
L_750	85002	79750	44.3b	1.24b	0.469b	45.1b	1.00	0.230b
SEM	4918	4866	5.40	0.092	0.027	5.48	-	0.014
*P*-Value	0.432	0.765	<0.001	<0.001	<0.001	<0.001	-	<0.001
D	0.715	0.734	0.416	0.361	0.479	0.397	-	0.570
L	0.030	0.217	0.779	0.001	<0.001	0.691	-	<0.001
D*L	0.532	0.528	0.916	0.562	0.081	0.918	-	0.419

CK, ensiling of sweet sorghum with 2.00 ml/kg fresh weight (FW) of distilled water at 650 (CK_650), 700 (CK_700), and 750 kg/m^3^ (CK_750) of density, respectively; L, ensiling of sweet sorghum with 2.00 g/t FW of lactic acid bacteria (LAB) inoculant and 2.00 mL/kg FW of distilled water at 650 (L_650), 700 (L_700), and 750 kg/m^3^ (L_750) of density, respectively. Values with different lowercase letters (a, b, and c) indicate significant differences among treatments (P < 0.05). LA, lactic acid; AA, acetic acid; D, silo density; L, inoculating LAB.

**Figure 1:**
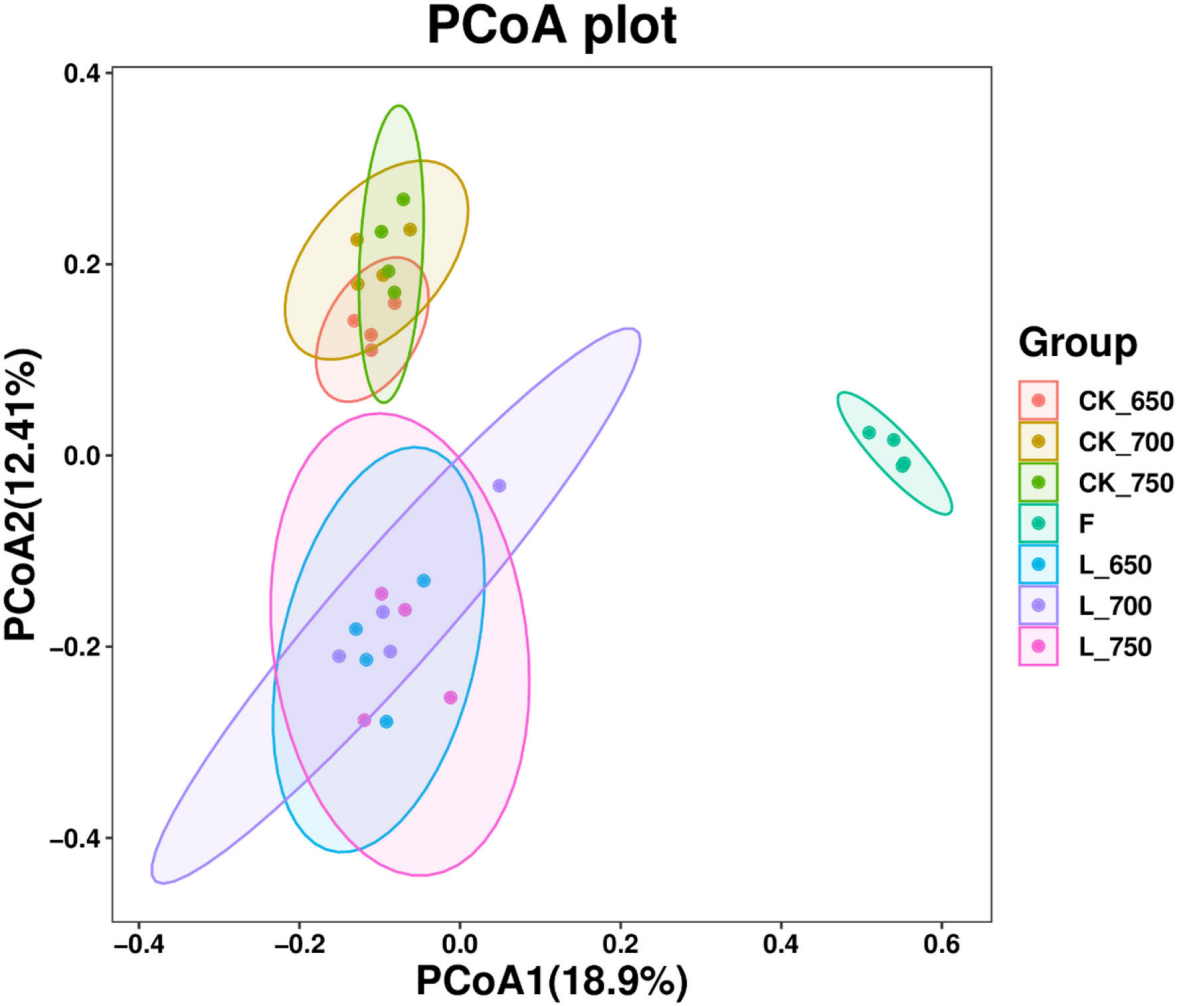
Principal coordinates analysis (PCoA) of bacterial community in sweet sorghum silages (*n* = 4). CK, ensiling of sweet sorghum with 2.00 ml/kg fresh weight (FW) of distilled water at 650 (CK_650), 700 (CK_700), and 750 kg/m^3^ (CK_750) of density, respectively; L, ensiling of sweet sorghum with 2.00 g/t FW of lactic acid bacteria (LAB) inoculant and 2.00 ml/kg FW of distilled water at 650 (L_650), 700 (L_700), and 750 kg/m^3^ (L_750) of density, respectively.

The fresh materials had clearly separated bacterial community from all silages, and the bacterial communities in CK_650, _700, and _750, and in L_650, _700, and _750 were clustered together, respectively, and clearly separated from each other (Figure 1).

**Figure 2:**
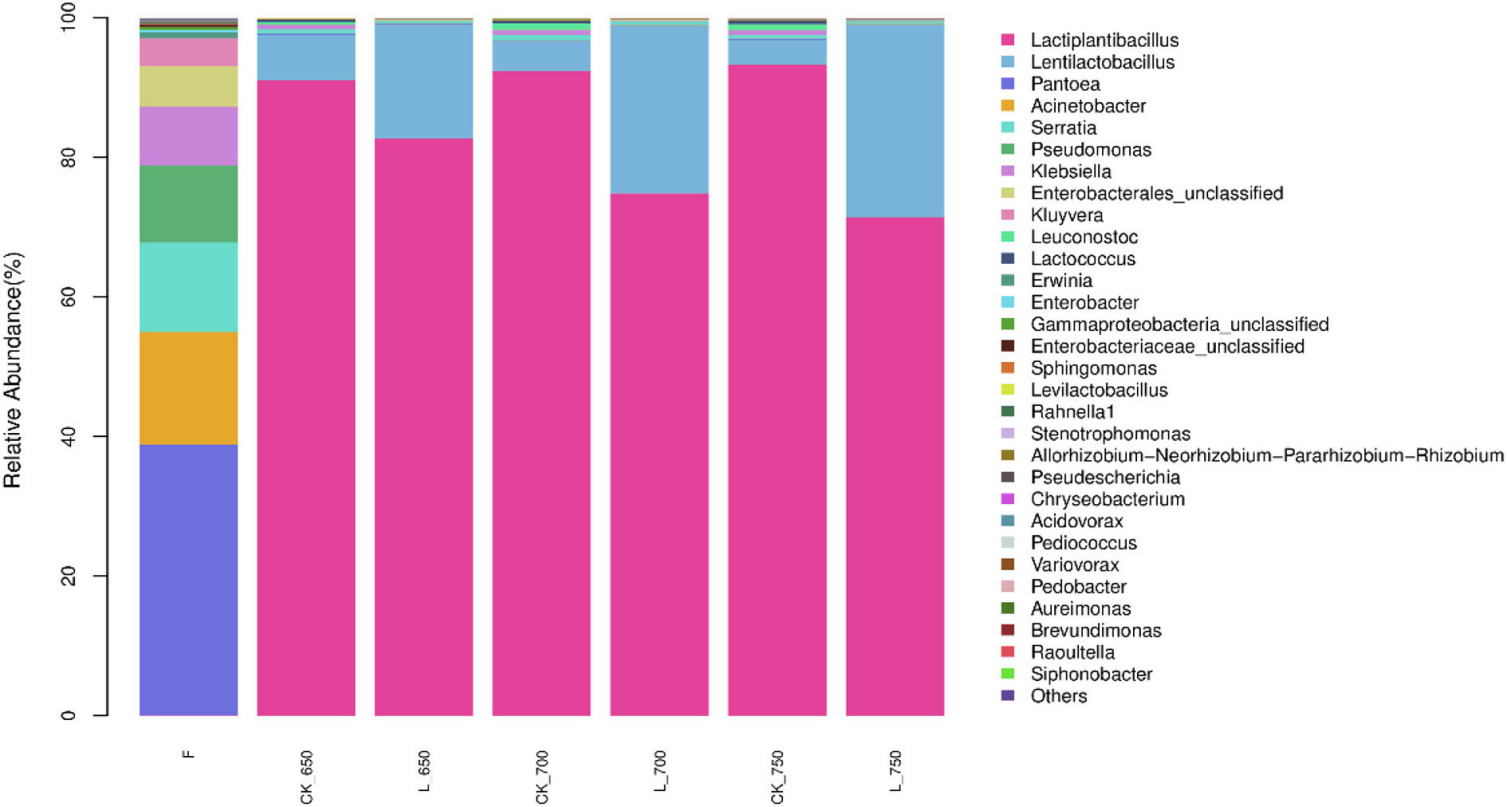
Relative abundance of bacterial community (genus level) in sweet sorghum silages (*n* = 4). CK, ensiling of sweet sorghum with 2.00 mL/kg fresh weight (FW) of distilled water at 650 (CK_650), 700 (CK_700), and 750 kg/m^3^ (CK_750) of density, respectively; L, ensiling of sweet sorghum with 2.00 g/t FW of lactic acid bacteria (LAB) inoculant and 2.00 mL/kg FW of distilled water at 650 (L_650), 700 (L_700), and 750 kg/m^3^ (L_750) of density, respectively.

The main bacterial genera in fresh materials were *Pantoea* (38.75%), *Acinetobacter* (16.19%), *Serratia* (12.81%), *Pseudomonas* (11.00%), *Klebsiella* (8.45%), and unclassified *Enterobacterales* (5.83%) (Figure 2). However, those genera in silages had <1% of abundance. *Lactiplantibacillus* was the most dominant bacterial genus in all silages, with an abundance from 71.39 to 93.27 %, followed by *Lentilactobacillus* (from 3.59 to 27.63%). Their total abundance was more than 96% in silage but <0.1% in fresh materials.

**Figure 3:**
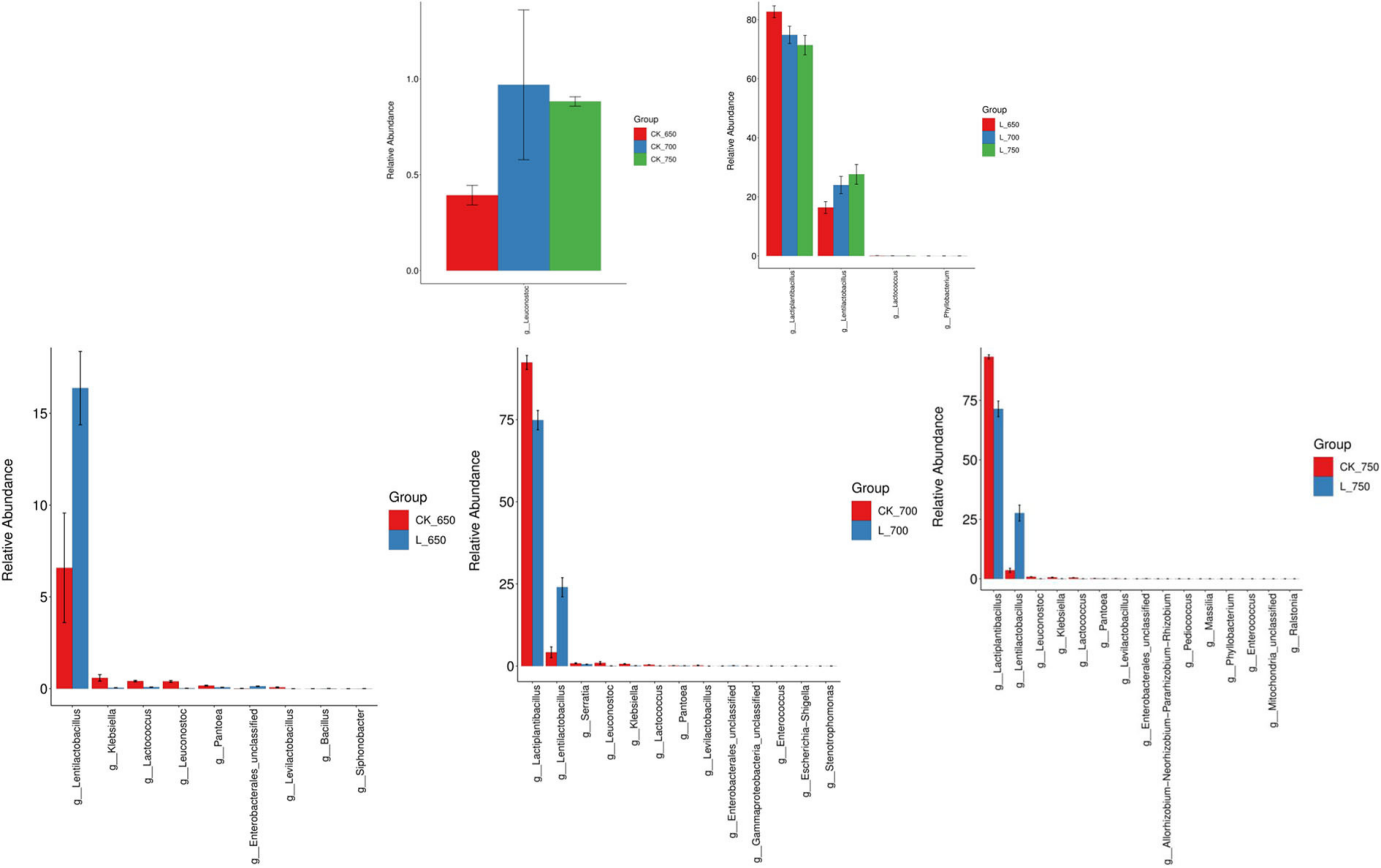
Difference in bacterial communities (genus level) in sweet sorghum silages (*n* = 4). CK, ensiling of sweet sorghum with 2.00 mL/kg fresh weight (FW) of distilled water at 650 (CK_650), 700 (CK_700), and 750 kg/m^3^ (CK_750) of density, respectively; L, ensiling of sweet sorghum with 2.00 g/t FW of lactic acid bacteria (LAB) inoculant and 2.00 mL/kg FW of distilled water at 650 (L_650), 700 (L_700), and 750 kg/m^3^ (L_750) of density, respectively.

CK_650 had less *Leuconostoc* abundance than CK_700 and _750 (*P* < 0.05) (Figure 3). L_650 had higher *Lactiplantibacillus* abundance but less *Lentilactobacillus* abundance than L_700 and _750 (*P* < 0.05). CK_700 and _750 had higher *Lactiplantibacillus* abundance than L_700 and 750, respectively (*P* < 0.05); moreover, CK_650, _700, and _750 had less *Lentilactobacillus* and higher *Klebsiella, Leuconostoc*, and *Lactococcus* abundance than L_650, _700, and 750, respectively (*P* < 0.05).

### Nutrition compositions of silages

L_750 had the highest DM content among all treatments (*P* < 0.05) (Table 6). L_700 had the lowest WSC among all treatments (*P* < 0.05), and CK_700 had lower WSC than CK_650 (*P* < 0.05). CK_650, _700, and L_700 had a higher ash concentration than L_650 and _750 (*P* < 0.05), and L_750 had a lower ash concentration than other treatments (except L_650) (*P* < 0.05). The DM content was mainly affected by silo density and inoculating LAB (*P* < 0.05). Inoculating LAB had a significant effect on the LA, AA, and ash concentrations (*P* < 0.05).

**Table 6:** Dry matter content (DM, g/kg) and nutritional compositions concentration (g/kg DM) in sweet sorghum silages (*n* = 4).

**Items**	**DM**	**CP**	**WSC**	**NDF**	**ADF**	**Ash**
CK_650	260b	58.5	222a	364	220	59.0a
L_650	271b	61.6	202ab	346	210	55.3bc
CK_700	260b	62.3	198b	336	206	58.5a
L_700	263b	62.0	180c	331	206	56.8a
CK_750	266b	60.2	214ab	350	211	58.1ab
L_750	286a	61.2	212ab	366	226	53.3c
SEM	3.36	1.33	5.05	11.6	7.35	0.760
*P*-value	<0.001	0.399	0.001	0.244	0.348	<0.001
D	0.002	0.149	<0.001	0.032	0.196	0.056
L	<0.001	0.146	0.001	0.780	0.801	<0.001
D*L	0.073	0.267	0.064	0.203	0.214	0.170

CK, ensiling of sweet sorghum with 2.00 mL/kg fresh weight (FW) of distilled water at 650 (CK_650), 700 (CK_700), and 750 kg/m^3^ (CK_750) of density, respectively; L, ensiling of sweet sorghum with 2.00 g/t FW of lactic acid bacteria (LAB) inoculant and 2.00 mL/kg FW of distilled water at 650 (L_650), 700 (L_700), and 750 kg/m^3^ (L_750) of density, respectively. Values with different lowercase letters (a, b, and c) indicate significant differences among treatments (P < 0.05). CP, crude protein; WSC, water-soluble carbohydrates; NDF, neutral detergent fiber; ADF, acid detergent fiber.

### Correlation between main bacterial genera and fermentation quality

**Figure 4:**
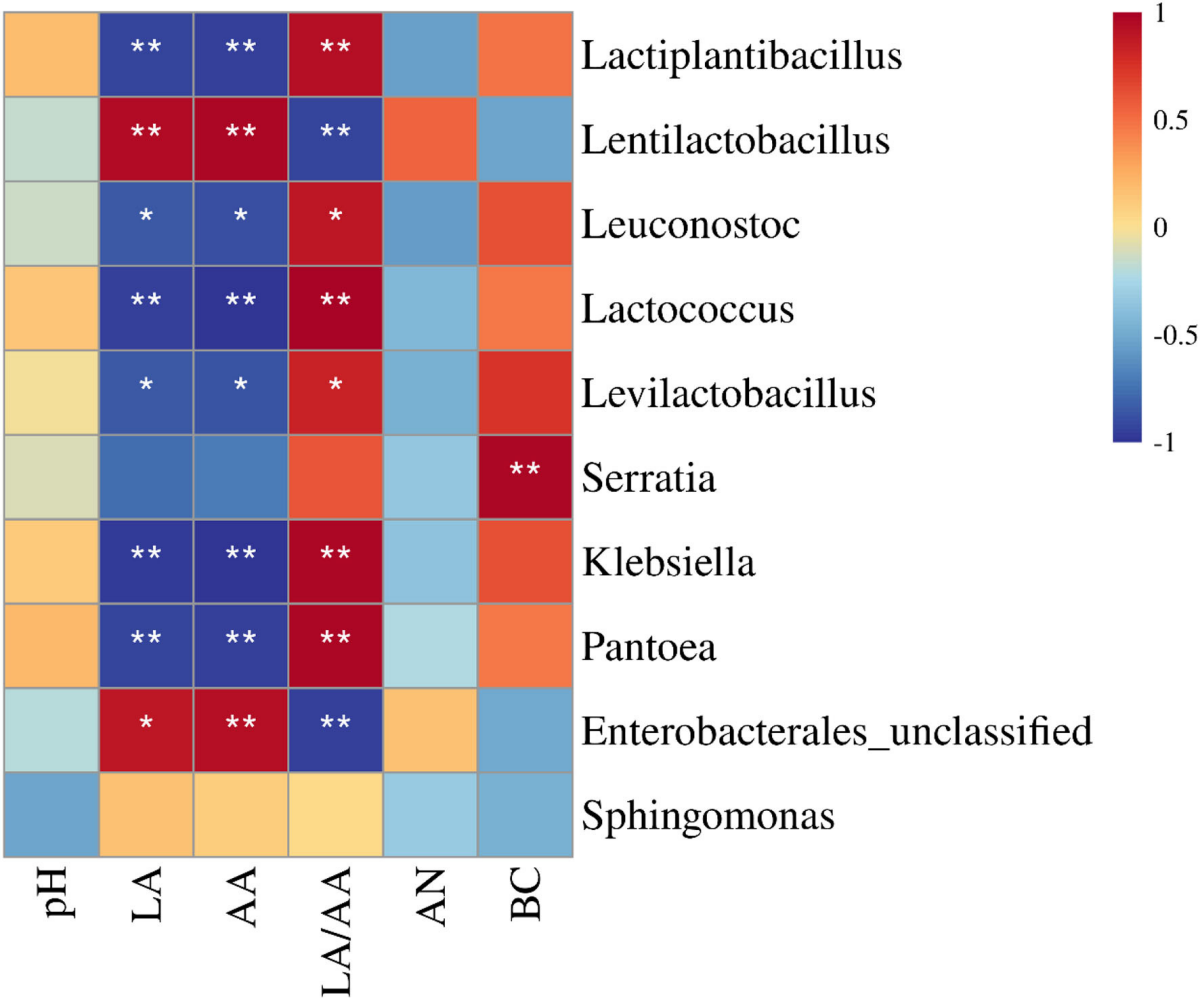
Correlation heatmap between main bacterial genera (top 10) and fermentation quality of sweet sorghum silages (*n* = 6). LA, lactic acid; AA, acetic acid; AN, ammonia nitrogen/total nitrogen; BC, buffering capacity. *p < 0.05 and **p < 0.01.

The LA and AA had a negative correlation with *Lactiplantibacillus, Leuconostoc, Lactococcus, Levilactobacillus, Klebsiella*, and *Pantoea* (*P* < 0.05), and a positive correlation with *Lentilactobacillus* and unclassified *Enterobacterales* (*P* < 0.05) (Figure 4). The LA/AA had an opposite relationship with aforementioned bacterial genera (*P* < 0.05). Moreover, the BC correlated positively with *Serratia* (*P* < 0.05).

## Discussion

### Characteristics of fresh sweet sorghum

In this study, the fermentation coefficient (FC = DM (%) + 8 WSC/BC) of fresh sweet sorghum was 38.7, which indicated that sweet sorghum was easily preserved as silage, because it has an FC of more than 35 (Alhaag et al., 2019), whereas the sweet sorghum pre-ensiling had 227 (mE/kg DM) of BC in the study (Table 1). Furthermore, previous studies also reported a BC of more than 250 (mE/kg DM) detected in fresh sweet sorghum (Sifeeldein et al., 2018; Alhaag et al., 2019), suggesting that in general, fresh sweet sorghum before ensiling has a relative high BC. The fresh forage contained sufficient WSC (327 g/kg DM) as a fermentation substrate (Table 1) for rapid propagation and growth of LAB during the fermentation process to overcome the high BC, which were reflected by the satisfactory fermentation quality of sweet sorghum silages with low pH (<4.0) and AN (<50 g/kg TN), and higher LA (>50 g/kg) (Table 3).

The main bacterial genera in fresh sweet sorghum were *Pantoea, Acinetobacter, Serratia, Pseudomonas, Klebsiella*, unclassified *Enterobacterales*, and *Kluyvera*, with 97.02% of total abundance (Figure 2). However, only two LAB genera (*Lactiplantibacillus* and *Lentilactobacillus*) were detected in fresh forage with 0.0675 and 0.0059% of abundances, respectively (Figure 2). Previous studies showed *Lactobacillus* had <1% of abundance in whole-plant corn, alfalfa, sweet sorghum, wheat, barley, and corn grain before ensiling (Gharechahi et al., 2017; Keshri et al., 2019; Carvalho-Estrada et al., 2020; Ren et al., 2021; Sun et al., 2021a,b,c; Na et al., 2022; Xia et al., 2022), indicating that the LAB genera, in general, present as minor taxa in forage pre-ensiling. The most abundant bacterial genus was *Pantoea* (38.75%) present in fresh sweet sorghum (Figure 2); a similar result was detected (35.8%) by Ren et al. (2021). However, Xia et al. (2022) reported *Bacillus* absolutely dominated the bacterial community (91.35%) in fresh sweet sorghum. The difference might be due to the difference in the geographical location and mowing period (McGarvey et al., 2013; Guan et al., 2020).

### Fermentation weight loss of silages

The losses of silage in bunker silo occurred commonly during filing, storage, and feed-out stages (Savoie and Jofriet, 2003), and anaerobic fermentation and releasing effluent strongly contribute to the losses during storage (Randby and Bakken, 2021). In this study, there were no visible effluents in silos; thus, the losses of sweet sorghum silage were caused mainly by anaerobic fermentation during storage. Fermentation-related losses in the silo are primarily from carbon dioxide production (gaseous losses) (Borreani et al., 2018). The FWL expressed in an FW basis can accurately estimate the gaseous losses of silage during fermentation because drying would result in the loss of fermentation products (organic acids and alcohols) (Samarasinghe et al., 2019).

The gaseous loss of silage during storage mostly resulted from the activities of heterofermentative LAB, enterobacteria, clostridia, and yeasts (Borreani et al., 2018). In this study, enterobacteria and yeast were present in the fresh material and silage, but clostridia were not detected (Tables 1, 4, Supplementary Figure S1), and *Lentilactobacillus buchneri*, as heterofermentative LAB, was one of two compositions of additives used in the study. Moreover, heterofermentative LAB dominates the initial fermentation process (the first 1 day) (Sun et al., 2021a), and *Lentilactobacillus* was the second largest bacterial group in silages, with abundance ranging from 3.59 to 27.63% (Figure 2). The activities of enterobacteria and yeast were inhibited by the pH <5.0 in silage (Samarasinghe et al., 2019), and the pH of sweet sorghum silage reduces to below 5.0 in the first 5 days of ensiling (Sifeeldein et al., 2018; Alhaag et al., 2019). Those indicated that in the study, the gaseous loss of sweet sorghum silage might be contributed by heterofermentative LAB, enterobacteria, and yeast growth during the initial fermentation process (the first 5 days) and caused by heterofermentative LAB activity during the rest of the fermentation period.

The intensity of initial fermentation in silage can be increased by inoculating LAB at ensiling (Sun et al., 2021a; Xu et al., 2021), which caused a rise in the initial FWL of silage (Samarasinghe et al., 2019). This explained that the FWL of inoculated silages was higher than that in uninoculated silages at 1 day after ensiling, respectively, although no difference was observed among all treatments (Table 2). The increasing intensity of initial fermentation caused less microbial activity during the late fermentation process (Samarasinghe et al., 2019; Sun et al., 2021a; Xu et al., 2021), resulting in the lower FWL of inoculated silages at 50 and 100 days of ensiling in the study (Table 2).

### Fermentation quality of silages

In the study, all silages showed satisfactory fermentation quality, as reflected by the low pH (<4.0) and AN content (<50 g/kg TN), and high LA concentration (>50 g/kg DM), and no propionic and butyric acids were detected (Table 3). Similar results have been reported by previous studies (Sifeeldein et al., 2018; Diepersloot et al., 2021). In general, inoculating homofermentative LAB at ensiling can increase the LA concentration in final silage, and inoculating heterofermentative LAB increases the AA concentration and reduces LA/AA in silage (Muck and Kung, 1997; Kung et al., 2018). *Lactiplantibacillus plantarum*, as one of two compositions in additives used in the study, might play an active role during the early fermentation process (Sun et al., 2021a) and produced more LA in inoculated silages (Table 3). *Lentilactobacillus buchneri*, as another composition of the additives, might play an important role during the late fermentation process (Herrmann et al., 2011; Blajman et al., 2018) and produced higher LA and AA in inoculated silages with sufficient WSC (Tables 3, 6). Compared with uninoculated silages, inoculated silages showed a higher *Lentilactobacillus* abundance with a positive correlation with LA and AA (Figures 2–4), which also explained those aforementioned observations. In the study, LA/AA in uninoculated silages was more than 6.7, and previous studies also reported higher LA/AA (5.4 and 7.8) in uninoculated sweet sorghum silages (Sifeeldein et al., 2018; Diepersloot et al., 2021). This might have resulted from the *Lactiplantibacillus* dominating the bacterial community (Figure 2) and the sufficient WSC in silage (Tables 1, 5) during the fermentation process. AN is part of the non-protein in silage and indicates the degree of silage preservation during fermentation (Thomas et al., 1980; Ke et al., 2015; Sun et al., 2021c). In the study, all silages were well preserved during the fermentation process, owing to the low level of AN (<50 g/kg TN) in silage, as shown in Kung et al. (2018). The AN concentration in uninoculated silages decreased with increasing silo density (Table 3). Previous studies also showed the decreasing trend of AN in the uninoculated sorghum silage, whole-plant corn silage, and whole-crop barley silage with increasing silo density (Sucu et al., 2016; Sun et al., 2021b), which might be contributed by the rapid fermentation of LAB under less oxygen content conditions in the silage with a high silo density during the early fermentation process (Tian et al., 2019). Previous studies reported that some LABs can produce the hydrolytic enzyme during the fermentation process to degrade protein in soybean, milk, and wheat flour (Rizzello et al., 2007; Tzvetkova et al., 2007; Aguirre et al., 2008; Stefańska et al., 2016). In the study, the AN content and the abundance of *Lentilactobacillus* increased in inoculated silages with rising silo density (Table 3 and Figure 2), and *Lentilactobacillus* showed a positive correlation with AN, although there was no significant difference (Figure 4), which indicated that *Lentilactobacillus* in inoculated silage might have the function of protein degradation of silage during fermentation. However, the protein degradation function of LAB in silage needs further study.

### Microbial counts and bacterial community

In this study, no difference in alpha diversity indexes was found among inoculated silages and among uninoculated silages, respectively, and the silo density did not mainly affect those indexes (Table 5). Similar results were also reported by previous studies in whole-crop barley silage and sorghum–sudangrass silage (Sun et al., 2021b; Bai et al., 2022), which suggested that increasing silo density does not alter the alpha diversity of the bacterial community in silage. Nevertheless, inoculating LAB at ensiling mainly affected and increased the Shannon, Simpson, Chao1, and Pielou e indexes of silage (Table 5), indicating that ensiling of sweet sorghum with LAB can improve the alpha diversity of the bacterial community in terminal silage. But previous studies reported the lower alpha diversity of the bacterial community in whole-plant corn silage and *Leymus chinensis* silage treated with LAB (Keshri et al., 2018; Xu et al., 2021). In the present study, inoculated silages and uninoculated silages had separated clustered bacterial communities in each group according to PCoA (Figure 1). Moreover, previous studies showed that with increasing silo density, bacterial communities were clustered together in whole-crop barley silage and sorghum–sudangrass silage (Sun et al., 2021b; Bai et al., 2022), which implied that the silage with different silo densities also has a similar bacterial community.

*Lactiplantibacillus* and *Lentilactobacillus* are named formerly as *Lactobacillus* (Zheng et al., 2020), and were dominant genera in the bacterial community of inoculated and uninoculated silages (total abundance of 98.89–99.11% and 97.73–98.06%, respectively) in the study (Figure 2). Similarly, previous studies reported that *Lactobacillus* had the most abundance in the bacterial community of uninoculated sorghum silage and sorghum–sudangrass silage (Forwood et al., 2019; Bai et al., 2022). However, other studies showed *Lactobacillus* presenting as a minor taxon in uninoculated sweet sorghum silage and sorghum silage (Ren et al., 2021; Forwood et al., 2022). These differences might be contributed by the different epiphytic microflora in the fresh forage (McGarvey et al., 2013; Guan et al., 2020). The inoculated silages had a higher LAB count and *Lactobacillaceae* than uninoculated silages for each silo density (Table 4 and Supplementary Figure S2), and inoculating LAB had a significant effect on the LAB count (Table 4), indicating that inoculating LAB at ensiling optimizes the bacterial community of sweet sorghum silage. The inoculated silages had higher *Lentilactobacillus* and lower *Lactiplantibacillus* than uninoculated silages for each silo density (Figures 2, 3), which might be related to the LAB additives used in the study and the characteristics of heterofermentative LAB. *Lentilactobacillus buchneri*, as heterofermentative LAB, was one of two compositions in the additives (*Lac. plantarum* and *Len. buchneri*). Moreover, *Lentilactobacillus* has strong adaptive to the niche of silage during the stable fermentation stage (Xia et al., 2022) and starts to activate in silage during the late fermentation process (Herrmann et al., 2011; Blajman et al., 2018). Ferrero et al. (2019) also showed a higher abundance of *Lentilactobacillus* in the stable fermentation phase than that in early fermentation stage. In the study, *Lactiplantibacillus* dominated the bacterial community (71.39–93.27%) in all silages at 100 days of ensiling (Figure 2); nevertheless, Xia et al. (2022) found *Lentilactobacillus* was the most abundant bacterial genus (>90%) in the mixture silage of *Sesbania cannabina* and sweet sorghum at 60 days of ensiling. The difference might have resulted from the different characteristics of the raw material. In the study, *Leuconostoc* in CK_650 had a lower abundance than that in CK_700 and _750 and presented as a minor taxon (<1%) in the bacterial community (Figures 2, 3). In the inoculated silages, with increasing silo density, *Lactiplantibacillus* had a decreasing abundance, but *Lentilactobacillus* had an increasing abundance, and the two genera had a total abundance of 96.58–99.08% (Figures 2, 3). Those showed that for sweet sorghum silage, increasing silo density has no effect on the bacterial community in uninoculated silage and affects the bacterial community in inoculated silage.

## Conclusion

The sweet sorghum pre-ensiling contained enough WSC and high BC. Increasing silo density and inoculating LAB reduced the FWL of sweet sorghum silage. The silage showed a satisfactory fermentation quality, and inoculating LAB increased the LA and AA concentrations and decreased the LA/AA. Inoculating LAB was the main factor affecting the bacterial community of the silage. *Lentilactobacillus* and *Lactiplantibacillus* presented as minor taxa in fresh sweet sorghum and dominated the bacterial community of the silage. Increasing silo density reduced *Lentilactobacillus* and raised *Lactiplantibacillus* in the inoculated silage. For each silo density, inoculating LAB also reduced *Lentilactobacillus* and raised *Lactiplantibacillus*.

## Data availability statement

The datasets presented in this study can be found in online repositories. The names of the repository/repositories and accession number(s) can be found in the article/Supplementary material.

## Author contributions

YX and JZ designed the study, funded, and supervised the experiments. HX and NW wrote the manuscript. HX, NW, NN, YZ, LS, HD, YF, and TW performed the experiments. HX, NW, YX, and JZ reviewed and edited the manuscript. HX, NW, NN, LS, and YZ analyzed the data. All authors reviewed the manuscript.

## Funding

This work was funded by the Strategic Priority Science and Technology Project of The Chinese Academy of Sciences (Category A) (Grant Number XDA26040201), National Natural Science Foundation of China (Grant Number 32160342), Science and Technology Project of Inner Mongolia (Grant Numbers 2020GG0049 and 2021GG0068), and Sustainable Development of Ecological Grassland of Inner Mongolia (Grant Number 2022CYZX04).

## Conflict of interest

The authors declare that the research was conducted in the absence of any commercial or financial relationships that could be construed as a potential conflict of interest.

## Publisher's note

All claims expressed in this article are solely those of the authors and do not necessarily represent those of their affiliated organizations, or those of the publisher, the editors and the reviewers. Any product that may be evaluated in this article, or claim that may be made by its manufacturer, is not guaranteed or endorsed by the publisher.

## Supplementary material

Supplementary Figure S1 The relative abundance of bacterial community (family level) in sweet sorghum silages (*n* = 4). CK, ensiling of sweet sorghum with 2.00 mL/kg fresh weight (FW) of distilled water at 650 kg/m^3^ (CK_650), 700 kg/m^3^ (CK_700), and 750 kg/m^3^ (CK_750) of density, respectively; L, ensiling of sweet sorghum with 2.00 g/t FW of lactic acid bacteria (LAB) inoculant and 2.00 mL/kg FW of distilled water at 650 (L_650), 700 (L_700), and 750 kg/m^3^ (L_750) of density, respectively.

Supplementary Figure S2 Difference in bacterial communities (family level) between inoculated and uninoculated silages for each silo density (*n* = 4). CK, ensiling of sweet sorghum with 2.00 ml/kg fresh weight (FW) of distilled water at 650 (CK_650), 700 (CK_700), and 750 kg/m^3^ (CK_750) of density, respectively; L, ensiling of sweet sorghum with 2.00 g/t FW of lactic acid bacteria (LAB) inoculant and 2.00 ml/kg FW of distilled water at 650 (L_650), 700 (L_700), and 750 kg/m^3^ (L_750) of density, respectively.

Supplementary Table S1 Difference in bacterial communities (family level) among inoculated silages (*n* = 4). L, ensiling of sweet sorghum with 2.00 g/t FW of lactic acid bacteria (LAB) inoculant and 2.00 ml/kg FW of distilled water at 650 (L_650), 700 (L_700), and 750 kg/m^3^ (L_750) of density, respectively.

## References

[B1] Adesogan A. T. Krueger N. Salawu M. B. Dean D. B. Staples C. R. (2004). The influence of treatment with dual purpose bacterial inoculants or soluble carbohydrates on the fermentation and aerobic stability of Bermudagrass. J. Dairy Sci. 87, 3407–3416. 10.3168/jds.S0022-0302(04)73476-1 15377619

[B2] Aguirre L. Garro M. S. Savoy de Giori G. (2008). Enzymatic hydrolysis of soybean protein using lactic acid bacteria. Food Chem. 111, 976–982. 10.1016/j.foodchem.2008.05.01835651055

[B3] Alhaag H. Yuan X. Mala A. Bai J. Shao T. (2019). Fermentation characteristics of *Lactobacillus Plantarum* and *Pediococcus* species isolated from sweet sorghum silage and their application as silage inoculants. Appl. Sci. 9, 1247. 10.3390/app9061247

[B4] Amer S. Seguin P. Mustafa A. F. (2012). Short communication: effects of feeding sweet sorghum silage on milk production of lactating dairy cows. J. Dairy Sci. 95, 859–863. 10.3168/jds.2011-4884 22281350

[B5] AOAC International (2005). Official Methods of Analysis, 18th Edn. Gaithersburg, MD: AOAC International.

[B6] Bai C. Pan G. Leng R. Ni W. Yang J. Sun J.. (2022). Effect of ensiling density and storage temperature on fermentation quality, bacterial community, and nitrate concentration of sorghum-sudangrass silage. Front. Microbiol. 13, 828320. 10.3389/fmicb.2022.828320 35250945 PMC8895230

[B7] Blajman J. E. Páez R. B. Vinderola C. G. Lingua M. S. Signorini M. L. (2018). A meta-analysis on the effectiveness of homofermentative and heterofermentative lactic acid bacteria for corn silage. J. Appl. Microbiol. 125, 1655–1669. 10.1111/jam.14084 30142700

[B8] Borreani G. Tabacco E. Schmidt R. J. Holmes B. J. Muck R. E. (2018). Silage review: factors affecting dry matter and quality losses in silages. J. Dairy Sci. 101, 3952–3979. 10.3168/jds.2017-13837 29685272

[B9] Cai Y. (1999). Identification and characterization of *Enterococcus* species isolated from forage crops and their influence on silage fermentation. J. Dairy Sci. 82, 2466–2471. 10.3168/jds.S0022-0302(99)75498-6 10575614

[B10] Carvalho-Estrada P. A. Fernandes J. Silva É. B. Tizioto P. Paziani S. F. Duarte A. P.. (2020). Effects of hybrid, kernel maturity, and storage period on the bacterial community in high-moisture and rehydrated corn grain silages. Syst. Appl. Microbiol. 43, 126131. 10.1016/j.syapm.2020.126131 32866836

[B11] Colombini S. Galassi G. Crovetto G. M. Rapetti L. (2012). Milk production, nitrogen balance, and fiber digestibility prediction of corn, whole plant grain sorghum, and forage sorghum silages in the dairy cow. J. Dairy Sci. 95, 4457–4467. 10.3168/jds.2011-4444 22818460

[B12] Diepersloot E. C. Pupo M. R. Ghizzi L. G. Gusmão J. O. Heinzen C. McCary C. L.. (2021). Effects of microbial inoculation and storage length on fermentation profile and nutrient composition of whole-plant sorghum silage of different varieties. Front. Microbiol. 12, 660567. 10.3389/fmicb.2021.660567 33927709 PMC8076742

[B13] Fernandes T. Paula E. M. Sultana H. Ferraretto L. F. (2020). Short communication: influence of sorghum cultivar, ensiling storage length, and microbial inoculation on fermentation profile, N fractions, ruminal *in situ* starch disappearance and aerobic stability of whole-plant sorghum silage. Anim. Feed Sci. Tech. 266, 114535. 10.1016/j.anifeedsci.2020.114535

[B14] Ferrero F. Piano S. Tabacco E. Borreani G. (2019). Effects of conservation period and *Lactobacillus hilgardii* inoculum on the fermentation profile and aerobic stability of whole corn and sorghum silages. J. Sci. Food Agric. 99, 2530–2540. 10.1002/jsfa.9463 30387150

[B15] Forwood D. L. Holman D. B. Chaves A. V. Meale S. J. (2022). Unsalable vegetables ensiled with sorghum promote heterofermentative lactic acid bacteria and improve *in vitro* rumen fermentation. Front. Microbiol. 13, 835913. 10.3389/fmicb.2022.835913 35633729 PMC9133931

[B16] Forwood D. L. Hooker K. Caro E. Huo Y. Holman D. B. Meale S. J.. (2019). Crop sorghum ensiled with unsalable vegetables increases silage microbial diversity. Front. Microbiol. 10, 2599. 10.3389/fmicb.2019.02599 31803152 PMC6872954

[B17] Gharechahi J. Kharazian Z. A. Sarikhan S. Jouzani G. S. Aghdasi M. Salekdeh G. H. (2017). The dynamics of the bacterial communities developed in maize silage. Microb. Biotechnol. 10, 1663–1676. 10.1111/1751-7915.12751 28696065 PMC5658587

[B18] Guan H. Shuai Y. Yan Y. Ran Q. Wang X. Li D.. (2020). Microbial community and fermentation dynamics of corn silage prepared with heat-resistant lactic acid bacteria in a hot environment. Microorganisms 8, 719. 10.3390/microorganisms8050719 32408707 PMC7285033

[B19] Herrmann C. Heiermann M. Idler C. (2011). Effects of ensiling, silage additives and storage period on methane formation of biogas crops. Bioresour. Technol. 102, 5153–5161. 10.1016/j.biortech.2011.01.012 21334882

[B20] Ke W. C. Yang F. Y. Undersander D. J. Guo X. S. (2015). Fermentation characteristics, aerobic stability, proteolysis and lipid composition of alfalfa silage ensiled with apple or grape pomace. Anim. Feed Sci. Technol. 202, 12–19, 10.1016/j.anifeedsci.2015.01.009

[B21] Keshri J. Chen Y. Pinto R. Kroupitski Y. Weinberg Z. G. Sela S. S. (2018). Microbiome dynamics during ensiling of corn with and without *Lactobacillus plantarum* inoculant. Appl. Microbiol. Biot. 102, 4025–4037. 10.1007/s00253-018-8903-y 29536147

[B22] Keshri J. Chen Y. Pinto R. Kroupitski Y. Weinberg Z. G. Sela S. S. (2019). Bacterial dynamics of wheat silage. Front. Microbiol. 10, 1532. 10.3389/fmicb.2019.01532 31354651 PMC6632545

[B23] Kung L. Shaver R. D. Grant R. J. Schmidt R. J. (2018). Silage review: interpretation of chemical, microbial, and organoleptic components of silages. J. Dairy Sci. 101, 4020–4033. 10.3168/jds.2017-13909 29685275

[B24] Li P. Shen Y. You M. Zhang Y. Yan J. Li D.. (2017). Effect of grape pomace on fermentation quality and aerobic stability of sweet sorghum silage. Anim. Sci. J. 88, 1523–1530. 10.1111/asj.12791 28485116

[B25] Logue J. B. Stedmon C. A. Kellerman A. M. Nielsen N. J. Andersson A. F. (2016). Experimental insights into the importance of aquatic bacterial community composition to the degradation of dissolved organic matter. ISME J. 10, 533–545. 10.1038/ismej.2015.131 26296065 PMC4817675

[B26] McGarvey J. A. Franco R. B. Palumbo J. D. Hnasko R. Stanker L. Mitloehner F. M. (2013). Bacterial population dynamics during the ensiling of *Medicago sativa* (alfalfa) and subsequent exposure to air. J. Appl. Microbiol. 114, 1661–1670. 10.1111/jam.12179 23521112

[B27] Muck R. E. Kung L. (1997). “Effect of silage additives on ensiling,” in Proceedigns of the Silage Field to Feedbunk North American Conference, Hershey, PA (Ithaca, NY: Northeast Regional Agricultural Engineering Service), 187–210.

[B28] Na N. Qili M. Wu N. Sun L. Xu H. Zhao Y.. (2022). Bacterial community and fermentation quality of ensiling alfalfa with commercial lactic acid bacterial additives. Front. Microbiol. 13, 836899. 10.3389/fmicb.2022.836899 35531295 PMC9073077

[B29] Orrico M. A. P. Vendramini J. M. B. Erickson J. Moriel P. Silveira M. L. A. Aguiar A. D.. (2020). Nutritive value and fermentation characteristics of silages produced from different sweet sorghum plant components with or without microbial inoculation. Appl. Anim. Sci. 36, 777–783. 10.15232/aas.2020-02027

[B30] Playne M. J. McDonald P. (1966). The buffering constituents of herbage and silage. J. Sci. Food Agr. 17, 264–268. 10.1002/jsfa.2740170609

[B31] Randby Å. T. Bakken A. K. (2021). Effect of acid based additive treatment of low dry matter grass crops on losses and silage quality in bunker silos. Anim. Feed Sci. Technol. 275, 114869. 10.1016/j.anifeedsci.2021.114869

[B32] Ren H. Sun W. Yan Z. Zhang Y. Wang Z. Song B.. (2021). Bioaugmentation of sweet sorghum ensiling with rumen fluid: fermentation characteristics, chemical composition, microbial community, and enzymatic digestibility of silages. J. Clean. Prod. 294, 126308. 10.1016/j.jclepro.2021.126308

[B33] Rizzello C. G. De Angelis M. Di Cagno R. Camarca A. Silano M. Losito I.. (2007). Highly efficient gluten degradation by lactobacilli and fungal proteases during food processing: new perspectives for celiac disease. Appl. Environ. Microb. 73, 4499–4507. 10.1128/AEM.00260-07 17513580 PMC1932817

[B34] Sabertanha E. Rouzbehan Y. Fazaeli H. Rezaei J. (2021). Nutritive value of sorghum silage for sheep. J. Anim. Physiol. Anim. Nutr. 105, 1–12. 10.1111/jpn.13548 33864304

[B35] Samarasinghe M. B. Larsen M. Johansen M. Waldemar P. Weisbjerg M. R. (2019). Effects of shredding on silage density and fermentation quality. Grass Forage Sci. 74, 244–253. 10.1111/gfs.12424

[B36] Savoie P. Jofriet J. C. (2003). “Silage storage,” in Agronomy Monograph, 42. Silage Science and Technology, eds Buxton D. R. Muck R. E. Harrison J. H. (Madison: American Society of Agronomy, Crop Science Society of America, Soil Science Society of America), 405–467. 10.2134/agronmonogr42.c9

[B37] Sifeeldein A. Wang S. Li J. Dong Z. Chen L. Kaka N. A.. (2018). Phylogenetic identification of lactic acid bacteria isolates and their effects on the fermentation quality of sweet sorghum (*sorghum bicolor*) silage. J. Appl. Microbiol. 126, 718–729. 10.1111/jam.14123 30288865

[B38] Stefańska I. Piasecka-Józwiak K. Kotyrba D. Kolenda M. Stecka K. M. (2016). Selection of lactic acid bacteria strains for the hydrolysis of allergenic proteins of wheat flour. J. Sci. Food Agric. 96, 3897–3905. 10.1002/jsfa.7588 26693837

[B39] Sucu E. Kalkan H. Canbolat O. Filya I. (2016). Effects of ensiling density on nutritive value of maize and sorghum silages. R. Bras. Zootec. 45, 596–603. 10.1590/S1806-92902016001000003

[B40] Sun L. Bai C. Xu H. Na N. Jiang Y. Yin G.. (2021a). Succession of bacterial community during the initial aerobic, intense fermentation, and stable phases of whole-plant corn silages treated with lactic acid bacteria suspensions prepared from other silages. Front. Microbiol. 12, 655095. 10.3389/fmicb.2021.655095 33841382 PMC8032959

[B41] Sun L. Jiang Y. Ling Q. Na N. Xu H. Vyas D.. (2021c). Effects of adding pre-fermented fluid prepared from lucerne or red clover on fermentation quality and *in vitro* digestibility of the ensiled wilting-forages. Agriculture 11, 454. 10.3390/agriculture11050454

[B42] Sun L. Na N. Li X. Li Z. Wang C. Wu X.. (2021b). Impact of packing density on the bacterial community, fermentation, and *in vitro* digestibility of whole-crop barley silage. Agriculture 11, 672. 10.3390/agriculture11070672

[B43] Thomas P. C. Chamberlain D. G. Kelly N. C. Wait M. K. (1980). The nutritive value of silages digestion of nitrogenous constituents in sheep receiving diets of grass-silage and grass silage and barley. Br. J. Nutr. 43, 469–479. 10.1079/BJN19800114 7417392

[B44] Tian J. Xu N. Liu B. Huan H. Gu H. Dong C.. (2019). Interaction effect of silo density and additives on the fermentation quality, microbial counts, chemical composition and *in vitro* degradability of rice straw silage. Bioresour. Technol. 297, 122412. 10.1016/j.biortech.2019.122412 31776105

[B45] Tudisco R. Morittu V. M. Musco N. Grossi M. Iommelli P. D'Aniello B.. (2021). Effects of Sorghum silage in lactating buffalo cow diet: biochemical profile, milk yield, and quality. Agriculture 11, 57. 10.3390/agriculture11010057

[B46] Tzvetkova I. Dalgalarrondo M. Danova S. Iliev I. Ivanova I. Chobert J.-M.. (2007). Hydrolysis of major dairy proteins by lactic acid bacteria from bulgarian yogurts. J. Food Biochem. 31, 680–702. 10.1111/j.1745-4514.2007.00137.x

[B47] Van Soest P. J. Robertson J. B. Lewis B. A. (1991). Methods for dietary fiber neutral detergent fiber and nonstarch polysaccharides in relation to animal nutrition. J. Dairy Sci. 74, 3583–3594. 10.3168/jds.S0022-0302(91)78551-2 1660498

[B48] Wang C. Sun L. Xu H. Na N. Yin G. Liu S.. (2021). Microbial communities, metabolites, fermentation quality and aerobic stability of whole-plant corn silage collected from family farms in desert steppe of North China. Processes 9, 784. 10.3390/pr9050784

[B49] Xia T. Wang T. Sun J. Shi W. Liu Y. Huang F.. (2022). Modulation of fermentation quality and metabolome in co-ensiling of *Sesbania cannabina* and sweet sorghum by lactic acid bacterial inoculants. Front. Microbiol. 13, 851271. 10.3389/fmicb.2022.851271 35401441 PMC8988063

[B50] Xu H. Sun L. Na N. Wang C. Yin G. Liu S.. (2021). Dynamics of bacterial community and fermentation quality in *Leymus chinensis* Silage treated with lactic acid bacteria and/or water. Front. Microbiol. 12, 717120. 10.3389/fmicb.2021.717120 34803939 PMC8595406

[B51] Zheng J. Wittouck S. Salvetti E. Franz C. M. A. P. Harris H. M. B. Mattarelli P.. (2020). A taxonomic note on the genus *Lactobacillus*: description of 23 novel genera, emended description of the genus *Lactobacillus* Beijerinck 1901, and union of *Lactobacillaceae* and *Leuconostocaceae*. Int. J. Syst. Evol. Micr. 70, 2782–2858. 10.1099/ijsem.0.004107 32293557

