# Supplementary figures and images for: Fermentation weight loss, fermentation quality, and bacterial community of ensiling of sweet sorghum with lactic acid bacteria at different silo densities
Source: Front Microbiol. 2022 Nov 14;13:1013913. doi: 10.3389/fmicb.2022.1013913 (PMC9703978; doi:10.3389/fmicb.2022.1013913)

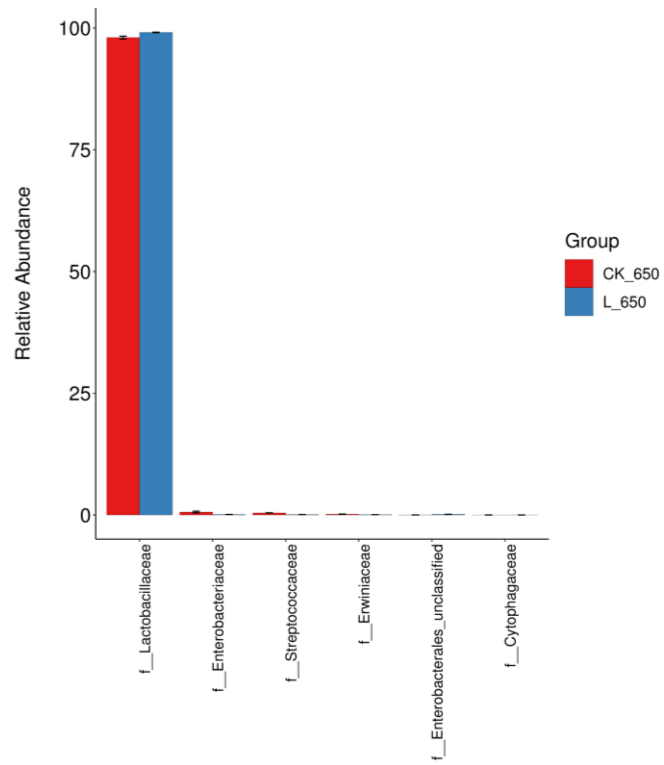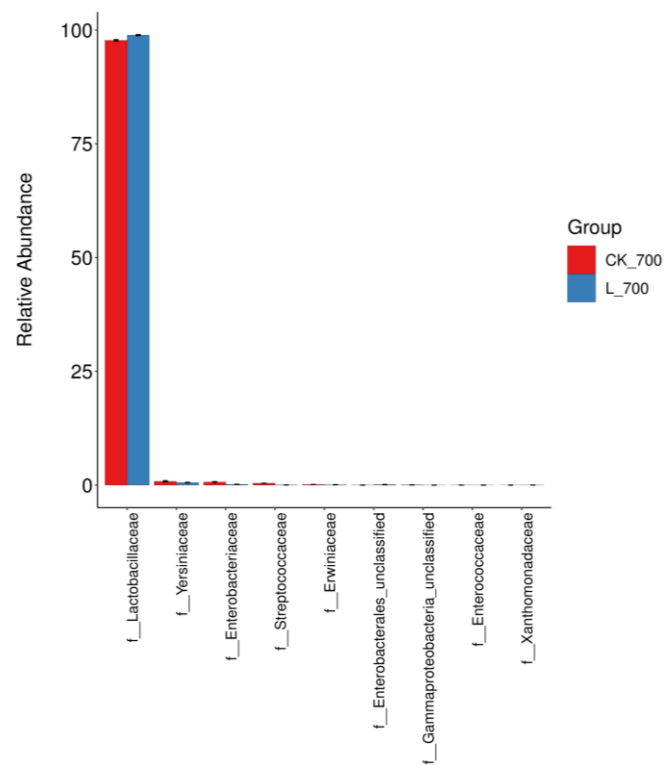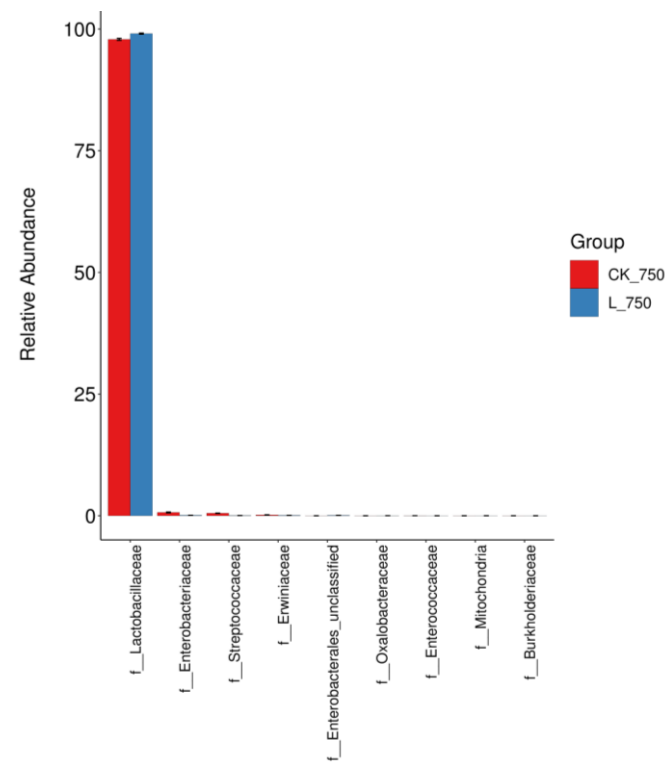

Supplement: Supplementary Figure S1 — The relative abundance of bacterial community (family level) in sweet sorghum silages (n = 4). CK, ensiling of sweet sorghum with 2.00 mL/kg fresh weight (FW) of distilled water at 650 kg/m3 (CK_650), 700 kg/m3 (CK_700), and 750 kg/m3 (CK_750) of density, respectively; L, ensiling of sweet sorghum with 2.00 g/t FW of lactic acid bacteria (LAB) inoculant and 2.00 mL/kg FW of distilled water at 650 (L_650), 700 (L_700), and 750 kg/m3 (L_750) of density, respectively. [file Image_1.pdf]

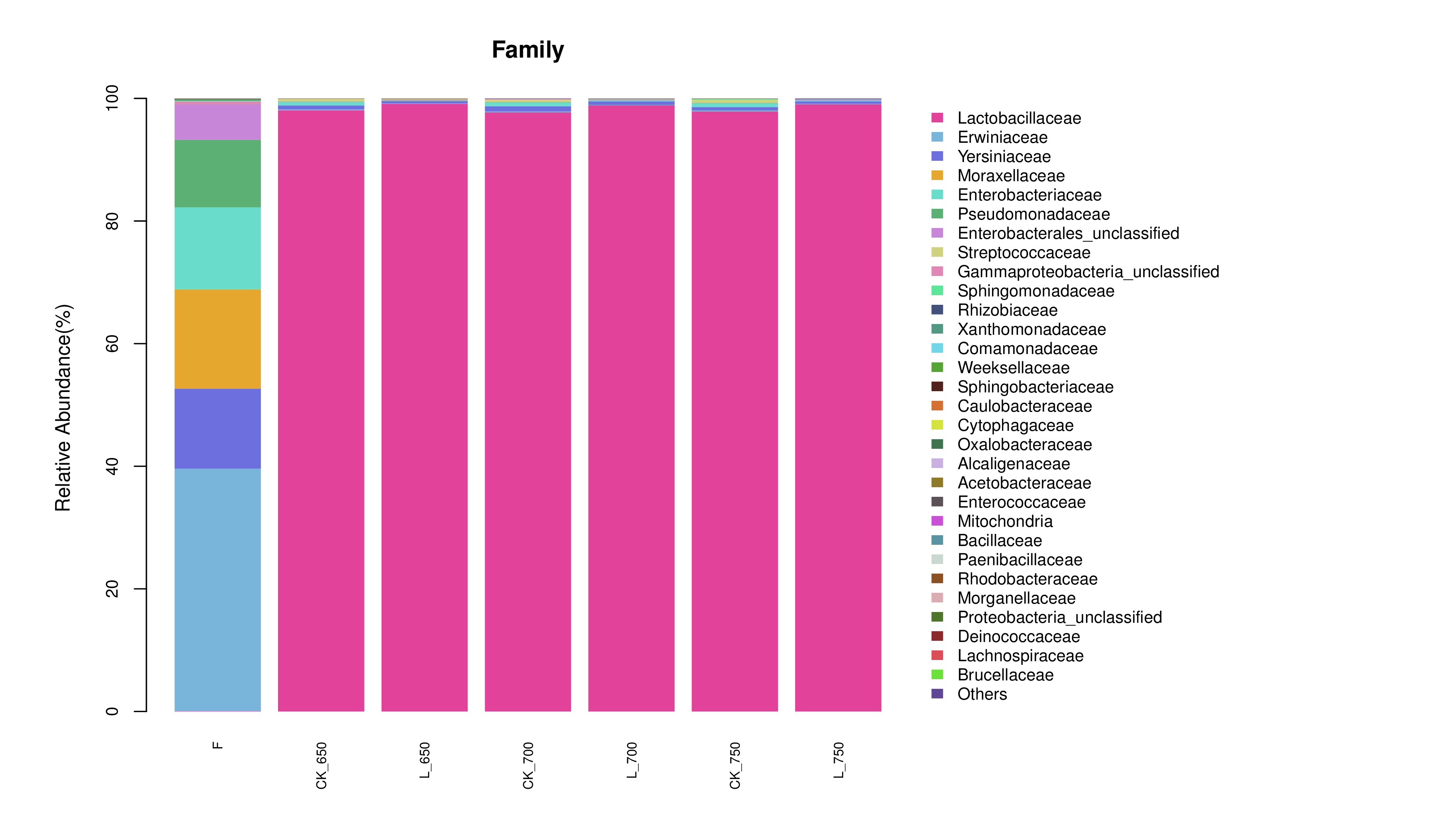

Supplement: Supplementary Figure S2 — Difference in bacterial communities (family level) between inoculated and uninoculated silages for each silo density (n = 4). CK, ensiling of sweet sorghum with 2.00 ml/kg fresh weight (FW) of distilled water at 650 (CK_650), 700 (CK_700), and 750 kg/m3 (CK_750) of density, respectively; L, ensiling of sweet sorghum with 2.00 g/t FW of lactic acid bacteria (LAB) inoculant and 2.00 ml/kg FW of distilled water at 650 (L_650), 700 (L_700), and 750 kg/m3 (L_750) of density, respectively. [file Image_2.png]
